# Prevalence of and factors associated with alexithymia among patients with chronic obstructive pulmonary disease in China: a cross-sectional study

**DOI:** 10.1186/s12890-023-02335-5

**Published:** 2023-01-30

**Authors:** Huaizhong Zhang, Yixuan Wang, Heqing Lou, Yanan Zhu, Zongmei Dong, Dong Dong, Peipei Chen, Xuan Zhu, Bi Chen, Pan Zhang

**Affiliations:** 1 Department of Psychiatry, Xuzhou Cancer Hospital, Xuzhou, 221000 Jiangsu China; 2 Department of Control and Prevention of Chronic Non-Communicable Diseases, Xuzhou Center for Disease Control and Prevention, 142 West Erhuan Road, Xuzhou, 221006 China; 3 Department of Respiratory Medicine of the Affiliated Hospital of Xuzhou Medical University, Xuzhou, China

**Keywords:** Chronic obstructive pulmonary disease, Alexithymia, Prevalence, Predictive factors, Cross-sectional, Investigation

## Abstract

**Background:**

Alexithymia is a common psychological disorder. However, few studies have investigated its prevalence and predictors in patients with chronic obstructive pulmonary disease (COPD). Therefore, we aimed to determine the prevalence and predictors of alexithymia in Chinese patients.

**Methods:**

This cross-sectional study included 842 COPD patients to assess the prevalence and predictors of alexithymia using the 20-item Toronto Alexithymia Scale (TAS-20). We used the Hospital Anxiety and Depression Scale (HADS) to assess anxiety and depression, the modified British Medical Research Council dyspnea Rating Scale (mMRC) to assess dyspnea, St. George's Respiratory Questionnaire (SGRQ) to assess quality of life, and the age-adjusted Charlson comorbidity index (ACCI) to assess comorbidities. Alexithymia-related predictors were identified using univariate and multivariate logistic regression analyses.

**Results:**

The prevalence of alexithymia in COPD patients was 23.6% (199/842). Multivariate analysis showed that age [odds ratio (OR) 0.886; 95% confidence interval (CI) 0.794–0.998], body mass index (OR 0.879; 95% CI 0.781–0.989), HADS-anxiety (OR 1.238; 95% CI 1.097–1.396), HADS-depression (OR 1.178; 95% CI 1.034–1.340), mMRC (OR 1.297; 95% CI 1.274–1.320), SGRQ (OR 1.627; 95% CI 1.401–1.890), ACCI (OR 1.165; 95% CI 1.051–1.280), and GOLD grade (OR 1.296; 95% CI 1.256–1.337) were independent predictors for alexithymia in patients with COPD.

**Conclusions:**

The prevalence of alexithymia was high in Chinese COPD patients. Anxiety, depression, dyspnea, quality of life, comorbidities, and disease severity are independent risk factors, and age and BMI are predictive factors for alexithymia in COPD patients.

## Background

Alexithymia is a multifaceted construct that is characterised by difficulties identifying one's feelings; difficulties describing one's feelings to others; and an externally focused, utilitarian cognitive style [1]. The term Alexithymia had been coined by Nemiah and Sifneos (1970) [1]. The 20-item Toronto Alexithymia Scale (TAS-20) is a self-reported questionnaire widly used to measure the severity of Alexithymia [2–9]. Hendrix et al. [5] defined alexithymia based on the following symptoms: (1) difficulty in identifying and describing emotions; (2) inability to effectively distinguish body feelings from emotions; (3) limited or lack of symbolic thinking ability; and (4) an extroverted way of thinking. The prevalence of alexithymia is 13.0% in the general population [6], and is much higher in patients with comorbidities, such as psoriasis, autism, and gastroenterological and hepatological diseases [7–9]. As for chronic respiratory diseases, a few studies have identified a high incidence rate for patients with alexithymia [10, 11].

COPD is a common, preventable, and treatable chronic disease that results from abnormal airway and/or alveoli caused by long-term exposure to toxic particles or gases. It is characterized by airflow restriction and persistent respiratory symptoms [12]. Globally, approximately 300 million people have COPD, and more than 3 million people die from it every year [13]. Accordingly, the prevalence of COPD will continue to rise in the next 40 years. By 2060, more than 5.4 million people are estimated to die of COPD and related diseases every year [14]. Therefore, COPD remains an important global public health issue. Patients with COPD often present with psychological adverse symptoms, such as depression, as a result of persistent airflow restriction and respiratory symptoms [15]. The incidence of alexithymia in patients with COPD is reportedly higher than that in healthy individuals [11].

Alexithymia aggravates the disease burden of patients [7], blunts the perception of symptoms [16], and decreases pulmonary function [17], moreover, it can interfere with treatment [18–20]. Therefore, the prevalence and factors related to alexithymia should be investigated in patients with COPD to establish a basis for decision-making with regard to the management of COPD.

The prevalence of COPD among adults aged at least 20 years is approximately 8.6%, and approximately 99.9 million patients with COPD were from China [21]. However, to date, only one study has evaluated the prevalence and related factors of alexithymia in patients with COPD in China [11]. Therefore, the purpose of our study was to assess the prevalence of and the factors associated with alexithymia in patients with COPD and to assess the effects of alexithymia on the quality of life.

## Methods

### Study setting and design

Xuzhou City in eastern China is a moderately developed city with a population of 10 million people and has a COPD registry system that covers all communities. According to this registry, 29,800 patients with COPD were registered in 2950 community health service stations or village clinics of 211 community health service centers or township hospitals in Xuzhou, suggesting that there are 10 patients with COPD in each community health service station or village clinic.

The cross-sectional study was conducted from December 2018 to June 2019 to determine the prevalence of alexithymia in patients with COPD and to identify its related factors. Multistage cluster sampling was performed for all 11 regions in the study area. First, using cluster random sampling, three community health service centers in urban areas or three township hospitals in rural areas were selected from each study region. Then, three community health service stations or three village clinics were respectively selected from each selected community health service center and township hospital. Finally, those with COPD who were registered in the health service stations or in the village clinics were selected. Data were collected by trained researchers through face-to-face interviews during regular patient visits to their respective health service stations or village clinics.

### Participants

**Fig. 1:**
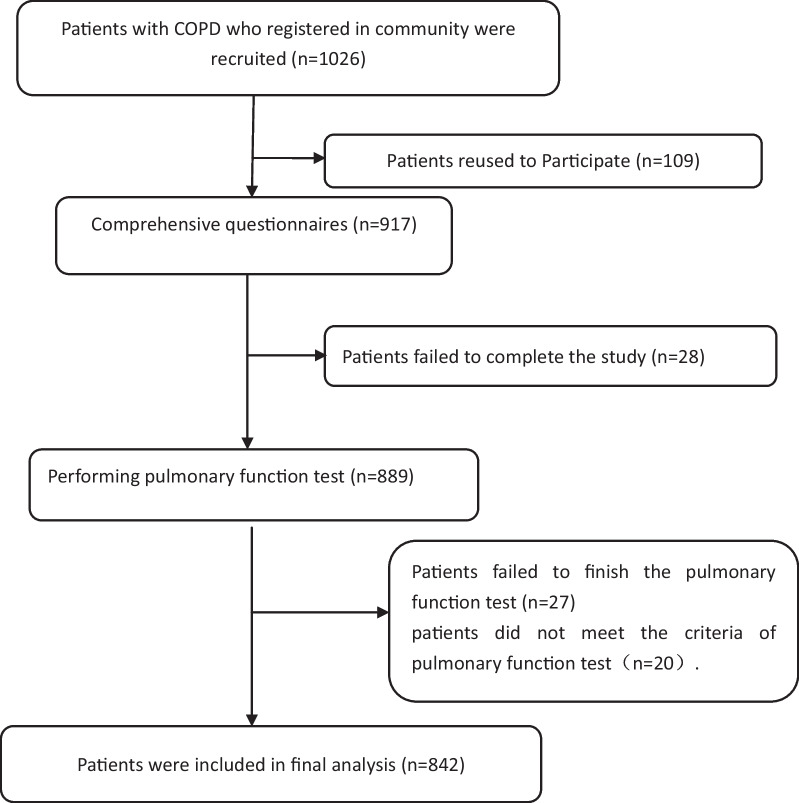
Flowchart of the study

Patients diagnosed with COPD according to the 2017 Global Initiative for Chronic Obstructive Lung Disease (GOLD) guidelines [12] were enrolled in our study. The inclusion criteria were stable condition, normal perception, normal communication skills, education level of primary school or above, and written informed consent. The exclusion criteria were acute exacerbation; presence of comorbid malignant tumors, active tuberculosis, severe liver disease, heart failure, myocardial infarction, cerebrovascular disease or serious orthopedic diseases; a history of alcoholism or psychotropic drug dependence; impaired cognitive functions, visual–auditory deficits, physical inability to accomplish the questionnaire without assistance; language communication disorder; and mental illness or use of any kind of psychotropic medication. Participants were excluded if there is any evidence of them to have other emotional or somatic illness. We determined the patients’ health conditions and psychotropic medication consuming according to the electronic medical record and prescription records from the electronic patients data within 6 months before the study. The sampling results are shown in Fig. 1.

The study protocol was approved by Xuzhou Center for Disease Control and Prevention (Approval No.: 201723). Written informed consent was obtained from all included participants. The procedures were in accordance with the standards of the ethics committee of Xuzhou Center for Disease Control and Prevention and the Declaration of Helsinki (1975, revised 2008), as well as the Strengthening the Reporting of Observational Studies in Epidemiology (STROBE) guidelines for observational studies [22].

### Variable collection and assessment

All patients were underwent the questionnaire survey and physical examination, including the following items.

#### Basic characteristics

Sex, age, years of education, occupation, smoking and drinking, body mass index (BMI), duration of disease, and comorbidities. Smoking refers to those who smoke one cigarette a day at the time of the survey and have been smoking for at least 6 months continuously or cumulatively in their life. Drinking refers to drinking at least once a week in the past year, regardless of season; drinking in the past is classified as not drinking. Comorbidities were assessed according to the age-adjusted Charlson comorbidity index (ACCI) [23], which included 18 comorbid conditions: (1) 1 point: myocardial infarction, congestive heart failure, peripheral vascular disease, cerebral vascular disease, dementia, connective tissue disease, ulcer disease, mild liver disease, or diabetes; (2) 2 points: hemiplegia, moderate/severe renal disease, diabetes with end-organ damage, any tumor, leukemia, or lymphoma; (3) 3 points: moderate/severe liver disease; and (4) 6 points: metastatic solid tumor or acquired immune deficiency syndrome. Age was scored as 1 point for each decade over age 40 years. Score of each item were accumulated as the total score.

#### Alexithymia

Alexithymia is measured by the Toronto Alexithymia Scale (TAS-20) which comprises 20 items rated on a 5-point Likert scale, ranging from 1 (totally disagree) to 5 (totally agree) [2]. A total score > 60 indicates alexithymic cases [3]. This scale has been translated in Chinese. The Chinese version of TAS-20 has been proved to have good reliability and validity [24, 25]. If the total score of TAS-20 is > 60, alexithymia is evaluated. Cronbach's α-coefficient in Chinese version of TAS-20 was 0.83, and the intraclass correlation coefficient (ICC) of test–retest reliability was 0.87. The criterion validity test showed that the correlation coefficients between the total score and each factor were 0.72–0.83, respectively [24].

#### Dyspnea

The modified British Medical Research Council dyspnea Rating Scale (mMRC) was used to assess dyspnea [26], with levels from 0 to 4. The higher the level, the more difficult it was to breathe. At present, the mMRC Dyspnea Scale is widely used in China.

#### Anxiety and depression

Depressive and anxiety symptoms were assessed using the hospital anxiety and depression scales (HADS) [27]. HADS consists of 14 items: seven items to assess anxiety (HADS-A) and seven items to assess depression (HADS-D). A score can range from 0 to 21:≥ 8 is defined as anxiety or depression, and ≤ 8 indicates that the subjects have no symptoms of anxiety or depression. The Chinese version Cronbach's alpha coefficients of the HADS-A and HADS-D subscales were 0.753 and 0.764, respectively [27].

#### Quality of life

St. George's respiratory questionnaire (SGRQ) [28] was used to evaluate the quality of life of patients. The questionnaire includes 54 items, and each item had a corresponding weight. The ratio between the sum of the weights of positive items and the total weight multiplied by 100 was the final score. The Chinese version correlation coefficients between the total score and the symptoms section score, the activity section score, and the impacts section score of SGRQ were 0.61, 0.79 and 0.97, respectively [28]. The higher the scores, the lower the quality of life.

#### Pulmonary function

Spirometry was measured using a portable spirometer (Medikro PRO, Finland) at health centers. Participants were asked whether they met the exclusion criteria of the pulmonary function test before the test. Lung function was expressed as the forced expiratory volume in 1 s as a percentage of the predicted value [FEV_1_ (% pred)]. Pulmonary function rank was categorized according to the GOLD guidelines based on the FEV_1_ (% predicted): GOLD 1, ≥ 80%; GOLD 2, 50–79%; GOLD 3, 30–49%; and GOLD 4, < 30% [12].

### Sample size calculation

At least 830 people were selected assuming an estimation prevalence of alexithymia of 25% in patients with COPD [11, 15] with an admissible error of 10%, power of 90%, and alpha of 0.05, and allowing for a refusal rate of 10%. Considering the exclusion criteria and those who refused to answer, eight communities were selected. With 10 patients with COPD in each station or clinic, considering the exclusion criteria and those who refused to answer, a total of 99 stations or clinics were selected.

### Primary and secondary outcomes

The primary outcome was the prevalence of alexithymia in patients with COPD. The secondary outcomes were related factors for alexithymia.

### Statistical analysis

Alexithymia-related factors in patients with COPD were analyzed using SPSS 17.0 (SPSS Inc., Chicago, IL, USA). The patients were divided into two groups according to the results of assessment with TAS-20. The COPD group comprised patients with a TAS-20 ≤ 60, and the alexithymia group included patients with an TAS-20 > 60. Measured data were tested for normality, and data conforming to the normal distribution are expressed as mean ± standard deviation $\left( {\overline{x} \pm s} \right)$, and the T-test was used for pairwise comparison. Data that were not normally distributed were expressed as median (interquartile range), and differences were compared by Mann–Whitney U tests. Qualitative data were expressed by frequency (percentage), and differences were analyzed by Pearson *χ*^2^ Test; Correlations between alexithymia and related factors were analyzed using Spearman’s correlation analysis.

Multivariate logistic regression analysis (dependent variables: 0, patients with COPD but without risk of alexithymia (TAS-20 ≤ 60); 1, patients with COPD and risk of alexithymia (TAS-20 > 60)) were used to assess the likelihood of risk of alexithymia versus no risk of alexithymia in patients with COPD, adjusted for (continuous), sex (male or female), educational level (lower than high school, high school or greater), BMI (continuous), smoking (yes or no), drinking habit (yes or no), duration of disease (continuous), HADS-A (yes or no), HADS-D (yes or no), mMRC (yes or no), SGRQ (yes or no), and GOLD grade (1, 2, 3, 4). MMRC, SGRQ, and ACCI scores were assigned a value of 1 if they were greater than the average and 0 if they were less than the average. Odds ratios (ORs) and 95%confidence intervals (CIs) were calculated. All tests were two sided, and *p* < 0.05 was considered statistically significant.

## Results

### Characteristics of study participants

A flowchart of the study design and process is indicated in Fig. 1. A total of 1026 patients with COPD registered in 99 village clinics (community health stations) were enrolled for this observational study. On the basis of the design principles, 184 subjects were excluded: 109 refused to participate, 28 failed to complete the study, 27 failed to finish the pulmonary function test, and 20 did not meet the criteria of pulmonary function test. Finally, 842 people with COPD were included in the analysis. The response rate was 82.1%. The mean age of participants was 61.69 ± 8.99 years, and 28.0% were women. The mean age of non-respondents was 63.5 ± 9.3 years, and 30.1% were women. Table 1 shows the general characteristics of participants according to alexithymia. There were no significant age or sex differences between participants and non-respondents (*p* > 0.05).

**Table 1:** General characteristics of participants between the two groups

Variables	Alexithymia group (n = 199)	Only COPD group (n = 643)	*χ*^2^/t	*p*
Gender (Male,%)	156 (78.39)	450 (69.98)	5.325	0.021
Age Y (mean ± SD)	60.15 ± 7.87	62.43 ± 10.99	− 2.718	0.007
Education levels (year) (mean ± SD)	8.91 ± 3.21	8.62 ± 3.53	1.034	0.301
BMI (Kg/m^2^,) (mean ± SD)	21.35 ± 2.17	21.88 ± 2.25	− 2.928	0.004
Smoking (n, %)	41 (20.60)	92 (14.31)	4.531	0.044
Drinking (n, %)	40 (20.10)	124 (19.28)	0.173	0.880
Duration of Disease (year) (mean ± SD)	10.25 ± 4.32	10.98 ± 4.41	− 2.050	0.041
FEV_1_ (% predicted) (mean ± SD)	56.08 ± 37.32	43.56 ± 29.51	4.896	< 0.001
HADS-A scores (mean ± SD)	9.93 ± 5.81	5.52 ± 3.37	13.328	< 0.001
HADS-D scores (mean ± SD)	9.71. ± 6.17	5.43 ± 3.34	12.613	< 0.001
mMRC scores (mean ± SD)	2.92 ± 2.31	2.03 ± 1.74	5.805	< 0.001
SGRQ scores (mean ± SD)	63.45 ± 9.76	48.33 ± 8.61	20.956	< 0.001
ACCI scores (mean ± SD)	4.5 ± 2.1	3.0 ± 1.8	9.862	< 0.001
GOLD grade (n, %)				
GOLD 1	17 (8.54)	93 (14.46)	14.137	0.003
GOLD 2	63 (31.66)	257 (39.97)		
GOLD 3	84 (42.21)	220 (34.21)		
GOLD 4	35 (17.59)	73 (11.35)		

Sex, smoking, drinking, and GOLD grade are presented as n; other values are the mean with SD; COPD, chronic obstructive pulmonary disease; BMI, body mass index; FEV1, forced expiratory volume in 1 s; HADS-A, Hospital Anxiety and Depression Scale for anxiety; HADS-D, Hospital Anxiety and Depression Scale for depression; mMRC, Modified British Medical Research Council; SGRQ, St. George's respiratory questionnaire; ACCI age-adjusted Charlson comorbidity index; GOLD, Global Initiative for Chronic Obstructive Lung Disease

### Prevalence and factors associated with alexithymia

A total of 199 patients had a total score of TAS-20 > 60, and 23.63% of all patients were diagnosed with alexithymia. The proportion of male sex, smoking, BMI, FEV_1_ (% predicted), HADS-A score, HADS-D score, mMRC score, SGRQ score, ACCI score, and GOLD grade in the alexithymia group was higher than those in the only-COPD group (*p* < 0.05). Conversely, age and BMI were significantly small, and duration of disease was relatively short in the alexithymia group compared with the only-COPD group (*p* < 0.05). Alexithymia was more significantly distributed in GOLD grades 3 and 4 than in GOLD grade 1 and 2 (*p* < 0.05). Data on education levels and drinking were not significantly different between groups (all *p* > 0.05).

### Correlations of alexithymia and related factors

Analysis of the relationships between alexithymia and variables revealed significant correlations between the groups in terms of age, BMI, FEV_1_(% predicted), HADS-A score, HADS-D score, mMRC score, SGRQ score, and ACCI score (all *p* < 0.05). Moreover, alexithymia was significantly negatively correlated with age, BMI and FEV_1_ (% predicted) (Table 2).

**Table 2:** Correlation analysis of alexithymia and risk factors

Variables	Age	BMI	FEV_1_(% predicted)	HADS-A scores	HADS-D scores	mMRC score	SGRQ scores	ACCI scores
r Values	− 0.234	− 0.251	− 0.175	0.376	0.342	0.175	0.441	0.181
*p* Values	< 0.001	< 0.001	0.001	< 0.001	< 0.001	< 0.001	< 0.001	< 0.001

*BMI* body mass index, *FEV*_*1*_ forced expiratory volume in 1 s, *HADS-A* Hospital Anxiety and Depression Scale for anxiety, *HADS-D* Hospital Anxiety and Depression Scale for depression, *mMRC* Modified British Medical Research Council, *SGRQ* St. George's respiratory questionnaire, *ACCI* age-adjusted Charlson comorbidity index

### Independent predictors for risk of alexithymia symptoms in COPD

Univariate logistic regression analysis was performed to determine the predictors of alexithymia in patients with COPD. COPD with alexithymia (TAS-20 scores > 60) was considered a dependent variable and assigned as 1, and TAS-20 scores ≤ 60 was assigned as 0. Sex, age, education levels, BMI, smoking, duration of disease, drinking HADS-A, HADS-D, mMRC, SGRQ, ACCI and GOLD grade were considered as independent variables and analyzed one by one by univariate logistic regression. The results showed that male sex, age, BMI, smoking, duration of disease, HADS-A HADS-D, mMRC, SGRQ, ACCI scores, and GOLD grade were associated with alexithymia in patients with COPD (Table 3) (*p* < 0.05).

**Table 3:** Results of univariate logistic regression to analyze factors associated with alexithymia in patients with COPD (TAS-20 > 60)

Variable	*β*	*χ*^2^	*P*	*OR*	95% CI
Male	0.440	5.325	0.020	1.554	1.022–2.359
Age	− 0.180	10.467	0.003	0.835	0.704–0.991
BMI	− 0.155	12.178	0.010	0.856	0.742–0.988
Smoking	0.438	4.531	0.045	1.551	1.010–2.377
Duration of disease	− 0.104	9.253	0.041	0.901	0.814–0.998
HADS-A	0.263	30.623	< 0.001	1.301	1.182–1.432
HADS-D	0.197	25.452	< 0.001	1.218	1.076–1.378
mMRC	0.315	32.251	< 0.001	1.371	1.255–1.496
SGRQ	0.623	36.894	< 0.001	1.865	1.457–2.386
ACCI	0.258	27.012	< 0.001	1.294	1.116–1.475
GOLD grade	0.304	27.495	< 0.001	1.356	1.212–1.515

Alexithymia (TAS-20 > 60) is considered as a dependent variable, whereas sex, age, BMI, smoking, drinking, duration of disease, FEV_1_% expected value, mMRC scores, HADS-A scores, HADS-D scores, SGRQ scores, and ACCI scores are independent variables. BMI, body mass index; FEV_1_, forced expiratory volume in 1 s; HADS-A, Hospital Anxiety and Depression Scale for anxiety; HADS-D, Hospital Anxiety and Depression Scale for depression; mMRC, Modified British Medical Research Council; SGRQ, St. George's respiratory questionnaire; ACCI age-adjusted Charlson comorbidity index; GOLD, Global Initiative for Chronic Obstructive Lung Disease

Multivariate logistic regression analysis was also used to analyze the risk factors for alexithymia in patients with COPD. Age, BMI, duration of disease, smoking, HADS-A, HADS-D, mMRC, ACCI scores, and GOLD grade were used as independent variables by forced entry method, after adjusting for sex, education levels, and drinking. Age, BMI, HADS-A, HADS-D, mMRC, SGRQ, ACCI scores, and GOLD grade were all identified as independent factors for alexithymia in patients with COPD. Higher HADS-A, HADS-D, mMRC, SGRQ, ACCI scores, and GOLD grade were independent risk factors for alexithymia in patients with COPD. Old age and high BMI are protective factors of alexithymia in patients with COPD (Table 4).

**Table 4:** Multivariate logistic regression analysis of predictors of alexithymia in patients with COPD

Variable	*β*	*χ*^2^	*P*	*OR*	95% CI
Age	− 0.121	5.553	0.045	0.886	0.794–0.988
BMI	− 0.129	2.811	0.048	0.879	0.781–0.989
HADS-A	0.213	17.358	< 0.001	1.238	1.097–1.396
HADS-D	0.163	16.602	< 0.001	1.178	1.034–1.340
mMRC	0.260	24.356	< 0.001	1.297	1.274–1.320
SGRQ	0.487	30.001	< 0.001	1.627	1.401–1.890
ACCI	0.172	20.131	< 0.001	1.165	1.051–1.280
GOLD grade	0.259	20.035	< 0.001	1.296	1.256–1.337

Alexithymia (TAS-20 > 60) is considered as a dependent variable and sex, age, BMI, smoking, drinking, duration of disease, FEV_1_% expected value, mMRC scores, HADS-A scores, HADS-D scores, SGRQ scores, and ACCI scores were independent variables. BMI, body mass index; FEV_1_, forced expiratory volume in 1 s; HADS-A, Hospital Anxiety and Depression Scale for anxiety; HADS-D, Hospital Anxiety and Depression Scale for depression; mMRC, Modified British Medical Research Council; SGRQ, St. George's respiratory questionnaire; ACCI age-adjusted Charlson comorbidity index; GOLD, Global Initiative for Chronic Obstructive Lung Disease

## Discussion

To the best of our knowledge, this cross-sectional study is the first to estimate the prevalence and possible predictors of alexithymia in a large sample of Chinese patients with COPD. The prevalence of alexithymia in patients with COPD was 23.63%, based on a threshold of TAS-20 > 60. Patients with COPD and alexithymia (TAS-20 > 60) had significantly higher BMI, FEV_1_ (% predicted), HADS-A score, HADS-A score, mMRC score, SGRQ score, ACCI score, and GOLD grade than those of patients with COPD without alexithymia (*p* < 0.05). Moreover, age, BMI, and duration of disease were relatively small or short, and the proportion of male sex was higher. However, education levels and drinking were not significantly different between the two groups. The SGRQ score was high in patients with alexithymia, indicating a poor quality of life. The prevalence of alexithymia was higher in patients with severe and very severe COPD than that in patients with mild and moderate COPD. These results suggested that patients with COPD and alexithymia had more severe clinical symptoms, worse pulmonary function and quality of life, and more obvious dyspnea, which is consistent with previous studies [11, 15].

The prevalence of alexithymia symptoms in patients with COPD was 23.63%, which was inconsistent with earlier studies [11, 15], notably lower than that reported in another Chinese study [11]. These differences might be due to different sampling methods, sample sizes, and recruitment objects. Han et al. [11] recruited patients with moderate COPD using a simple individual sampling in an outpatient department of a comprehensive hospital. In contrast, the present study enrolled patients with COPD registered in the communities by multistage cluster sampling. Moreover, Han et al. [11] assessed 53 cases only, while we analyzed 843 cases, therefore indicating the reliability of the results of the present study with regard to the prevalence of alexithymia in patients with COPD. However, the prevalence of alexithymia symptoms in patients with COPD was higher in the present study than that in the study of Tselebis et al. [13]. The discrepancy might be due to the different recruits and different sampling methods, or differences in social culture. Compared with Westerners, Chinese people are reluctant to express their inner psychology and emotions [29]. The results showed that the prevalence of alexithymia in patients with COPD in China was higher than that of Western patients with COPD. Moreover, general practitioners in Chinese communities should screen patients with COPD for alexithymia to improve their quality of life.

In the present study, correlation analysis showed that alexithymia in patients with COPD were negatively correlated with the male sex and higher FEV_1_ (%predicted) value, which were in line with a previous study that showed negative relationships between alexithymia and higher FEV_1_ (%predicted) value and that male patients with COPD are prone to alexithymia [11]. However, these results were inconsistent with those of Tselebis et al. [15]. The association of alexithymia with the male sex in the general population has been confirmed by several studies [5, 30, 31]. Men had more symptoms of alexithymia than women, which might be attributable to the women’s role of being more emotional and extroverted than men [32, 33]. A higher TAS-20 score (> 60) was not only associated with a lower FEV_1_ (%predicted) value, but related to the severity of the disease in our study, which was consistent with those in the literature [10, 19]. Among patients with asthma, patients with alexithymia cannot perceive dyspnea in a time and clearly, which underestimates the severity of asthma deterioration and increases the risk of fatal or near-fatal asthma attack [10]; this disease also influences treatment effects [19]. Therefore, patients with COPD and alexithymia may not be able to clearly and timely perceive their real condition; may underestimate the severity of the condition; and may be unwilling to seek medical treatment. However, diagnosis may still be inaccurate or treatment might be inappropriate owing to the lack of communication capabilities to express their feelings.

Results of our multiple regression analysis in this study also revealed that age and BMI were independent predictors for alexithymia in patients with COPD. COPD is a chronic consumptive disease, and patients tend to have a low BMI, especially those in the advanced stages [34], while those with relatively large BMI might be in a relatively good physical condition, so they might have fewer psychological disorders. Age is a predictive factor for alexithymia in patients with COPD, as younger patients are unwilling to report their condition or share their feelings [35].

Consistent with previous research findings, our results showed that alexithymia was highly correlated with the degree of anxiety and depression in patients with COPD [15, 36, 37]. Anxiety and depression in patients with COPD not only aggravate the disease burden but also more likely lead to early death [38]. The results of our study showed that anxiety and depression were independent risk factors for alexithymia in patients with COPD. Therefore, medical staff should consider the existence of alexithymia when treating psychological diseases of patients with COPD.

Another important finding of the study is that some clinical outcomes are significantly associated with an increased likelihood of alexithymia against patients with COPD.

First, the high mMRC scores were a negative factor of alexithymia, which is consistent with that the previous study that reported the association of shortness of breath with alexithymia despite several adjustments for physical and mental health [39]. This may be the intermediary effect of anxiety and depression. Moreover, both have been associated with the severity of dyspnea, even after controlling for clinical respiratory status [40].

Second, patients with COPD tended to have more symptoms of alexithymia when SGRQ score was high. Alexithymia negatively impacts the health-related quality of life of the patients, regardless of the instrument used to define quality of life [41, 42] and in particular, patients with severe asthma [37, 43]. Even after the timely resolution of depression, alexithymia still reduces the quality of life of patients with obstructive sleep apnea [13]. Our results add new evidence to this view.

Third, a higher ACCI score acted as a risk factor for alexithymia in patients with COPD. A higher age-adjusted CCI score was associated with higher mMRC score and more severe lung function impairment [44]. Comorbidity is closely related to the GOLD score and predicted all-cause mortality of patients with COPD [45, 46]. This means that patients with COPD experience more unspeakable suffering, resulting in a high incidence of alexithymia.

One of the main differences between this and previous studies on alexithymia in patients with COPD is the statistical model used to adjust the predictors. Additionally, the strengths of this study included the use of a community-based multistage sampling design, large sample size, and random cluster sampling.

However, this study has some limitations. First, there is no control group. Second, we use a cross-sectional design; therefore, the reported results should only be interpreted as an association between alexithymia and the selected variables, where causality cannot be established. Third, alexithymia and other clinic outcomes were identified based on self-reported questionnaire data; therefore, the data were subject to recall problems and misunderstanding of the question, leading to information bias. Fourth, a large number of patients were excluded, resulting in selection bias. Finally, patients with less than primary school education were excluded, which also resulted in selection bias.

## Conclusion

Our study revealed a high prevalence of alexithymia symptoms in a large sample of Chinese patients with COPD. Patients with COPD and alexithymia have worse pulmonary function, poor quality of life, and more severe symptoms of dyspnea. Male sex, age, BMI, HADS-A, HADS-D, mMRC, SGRQ, ACCI and GOLD grade were independent factors for alexithymia in patients with COPD. Patients with severe COPD have a higher risk of alexithymia. Despite the high prevalence of alexithymia and poor quality of life in patients with COPD, alexithymia is still not included in the latest expert consensus on the management of COPD comorbidity [47]. Patients with alexithymia are unable to describe their feelings because of unclear expression, thus reducing communication and interaction with doctors, and deteriorating their condition. Therefore, the results suggest that medical institutions should improve the follow-up of patients with COPD and alexithymia and consider the factors of alexithymia when formulating a treatment plan of COPD that includes not only drug treatment but also appropriate psychotherapy.

## Data Availability

All data relevant to the given manuscript have been stored in a separate file, which can be made freely available to external investigators upon request. If someone wants to request the data from this study, please contact to correspondence to Pan Zhang. Email: Pan Zhang: xzzhangpan@126.com.
